# The Immunoregulatory Effect of Aconite Treatment on H22 Tumor-Bearing Mice via Modulating Adaptive Immunity and Natural Killer-Related Immunity

**DOI:** 10.1155/2023/1481114

**Published:** 2023-01-30

**Authors:** Huan Wang, Xiu zhong Qi, Wentao Jia, Jiahui Yu, Kangdi Yang, Xu Zhang, Lina Wang

**Affiliations:** ^1^Department of Traditional Chinese Medicine, Naval Medical University, No. 168 Changhai Road, Shanghai 200433, China; ^2^Unit 71345 of the Chinese People's Liberation Army, Zibo 255000, China; ^3^Department of Traditional Chinese Medicine, Qingdao Special Service Sanatorium of PLA Navy, No. 27 Xianggang West Road, Qingdao 266071, China; ^4^Department of Traditional Chinese Medicine, Chinese PLA General Hospital, No. 28 Fuxing Road, Beijing 100853, China

## Abstract

Hepatocellular carcinoma (HCC) is the most common type of primary liver cancer and, in its advanced stages, has a 5-year survival rate of only 3% to 5%. Despite novel mechanisms and treatments being uncovered over the past few years, effective strategies for HCC are currently limited. Previous studies have proven that aconite can suppress tumor growth and progression and prevent the recurrence and metastasis of multiple cancers, but the underlying molecular mechanisms are largely unknown. In this study, different doses of aconite were applied to mice bearing subcutaneous HCC tumors. It was found that aconite had a therapeutic effect on H22 tumor-bearing mice in a dose-dependent manner by reducing tumor volumes and prolonging survival times, which could be attributed to the immunoregulatory effect of aconite. Furthermore, results showed that high-dose administration of aconite could enhance adaptive immunity and natural killer (NK) cell-mediated immunity by regulating the secretion of interferon-*γ*, upregulating T cells and NK cells, and modulating the expression of the NK cytotoxicity biomarker CD107a and the inhibitory receptor TIGIT. This study revealed a novel mechanism through which aconite exerts antitumor effects, not merely through apoptosis induction pathways, providing more sound evidence that aconite has the potential to be developed into an effective anti-HCC agent.

## 1. Introduction

Primary liver cancer is one of the most fatal malignancies, causing more than 2,814,000 deaths in China annually [1, 2]. Hepatocellular carcinoma (HCC) is the most common type of primary liver cancer, accounting for 75% to 85% of all cases [2]. Although novel mechanisms and treatments have increasingly been uncovered over the past decade, effective strategies for HCC are currently limited, leading to a 5-year survival rate of only 3% to 5% in advanced HCC [3, 4]. Currently, the 5-year survival rates of HCC patients who receive hepatectomy are still below 60%, with a high recurrence rate of 60% to 80% [3]. The dismal cure rates of HCC can be attributed not only to the aggressiveness of HCC but also to the poor overall status of patients, especially impaired tumor immunity. Currently, the immunological status of patients with HCC is increasingly regarded as the most critical factor that affects cancer progression. In the immune microenvironment of HCC patients, cells including CD4^+^/CD8^+^ T cells, dendritic cells (DCs), and natural killer (NK) cells can promote immune response and exert antitumor effects, which contributes to effective immunological surveillance and mediates residual tumor cell eradication after radical surgery [5]. Nevertheless, most HCC patients present with impaired immunological status, which can result in failed tumor eradication and, subsequently, HCC progression.

Several immunotherapy schemes have been promoted, including tumor vaccines, immune modulators, adoptive immune cell transfers, and immune checkpoint inhibitors. High rates of drug resistance and severe immune-related adverse events remain the major obstacles to the development of such immune-targeting therapies. The efficacy of immunotherapy in liver cancer is relatively low compared with other cancers due to the peculiar immunotolerance characteristics of the liver [6]. Serving as antitoxic, cytotoxic, and fitness-enhancing agents, traditional Chinese medicines (TCMs) have long been proven to be effective in alleviating clinical symptoms, improving quality of life and immune function, and prolonging the survival of cancer patients. The liver-specific immunotolerance that leads to tumor escape is hard to reverse with single-target treatments [6]. However, the multitarget characteristics of TCM compounds may provide new ideas and methods for HCC immunotherapy.

In TCM theory, the immunodeficient condition of patients with advanced HCC can be recognized as “Yang-deficiency and Yin-excessiveness,” which can be characterized by symptoms including drowsiness, fatigue, edema, chilliness in the abdomen and limbs, and diarrhea. One of the principles of TCM treatment is “Yang-warming.” As a typical herb used in Yang-warming methods, *Aconitum carmichaeli* Debx. (aconite) has been shown to have clinical efficacy in treating advanced HCC [7]. Studies have shown that aconite can suppress tumor growth and progression, prevent recurrence and metastasis of multiple cancers, and alleviate pain and other symptoms, including diarrhea and chilliness, in patients undergoing chemotherapy [8, 9]. These antitumor effects of aconite can be attributed to inducing apoptosis, inhibiting inflammation, and suppressing epithelial-to-mesenchymal transformation [10, 11]. Nevertheless, few studies on the immunoregulation effect of aconite in HCC have been reported.

This study was designed to evaluate the effect of aconite on the immune status of mice with subcutaneous HCC tumors. Our results showed that aconite had a therapeutic effect on H22 tumor-bearing mice that could be attributed to its immunoregulatory effects, involving adaptive and NK cell-mediated immunity. This study revealed a novel mechanism through which aconite exerts an antitumor effect, not merely through apoptosis induction pathways, providing evidence that aconite has the potential to be developed into an effective anti-HCC agent.

## 2. Materials and Methods

All animal studies were conducted in accordance with protocols from the Animal Care and Use Committee of the Naval Medical University. Also, all possible steps were taken to avoid animal suffering at each stage of the experiment.

### 2.1. Preparation of Aconite Decoction

Aconite herbs were purchased from the Pharmacy of TCM, Changhai Hospital, affiliated with the Naval Medical University, Shanghai, China. The original solution of aconite decoction was prepared as follows: (1) 100 g of crude aconite was immersed and boiled in 1000 mL of diluted water for 2 h and then filtered; (2) this was repeated again; and (3) the two decoctions were mixed and concentrated to 100 mL. Aliquots of the original solution (1 g/mL) were stored at 4°C for further experiments.

### 2.2. HCC Xenografts and Grouping of Mice

In total, 50 4-week-old male BALB/c mice were purchased from SLAC Co., Ltd. (Shanghai, China). The mice were housed in a specific pathogen-free animal laboratory of Changhai Hospital, with a 12 h light/dark cycle lighting condition, 15×/h air ventilation, and 21 ± 1°C controlled temperature. During the experimental period, all mice had free access to standard fodder and distilled water, which were provided by SLAC Co., Ltd. and the animal laboratory. H22 cells (kindly provided by the TCM Laboratory of Changhai Hospital, Shanghai) were cultured for three passages and confirmed to be uncontaminated by a mycoplasma test. Then, 1 mL of H22 cells (1 × 10^7^ cells/mL) were intraperitoneally injected into BALB/c mice. After 5 days, the ascitic fluid was drawn from the abdominal cavity and intraperitoneally reinjected into another healthy BALB/c mice. When the mice exhibited abdominal bulging, 5 mL of ascites (flaxen-colored) were collected and diluted with saline solution to a concentration of 1 × 10^7^ cells/mL suspension.

Except for mice in the blank group, which were inoculated with saline solution, all other mice were subcutaneously inoculated in the left inguinal region with the aforementioned H22 cell suspension (0.2 mL/mice). This was followed by a 3-week treatment, starting the day after H22 inoculation, with saline solution or aconite in different concentrations (0.2 mL/20 g body weight per day, intragastric administration (i.g.)). The mice were then divided into five groups (10 mice/group): blank (no tumor grafted; saline solution i.g.), subcutaneous tumor-bearing model mice (saline solution i.g.), low dose (LD; aconite at 0.125 g/mL i.g.), medium dose (MD; aconite at 0.25 g/mL i.g.), and high dose (HD; aconite at 0.5 g/mL i.g.). During the administration period, the general condition and body weight dynamics of the mice were recorded daily. Mice were sacrificed after 3 consecutive weeks of aconite administration, and the survival times of mice in each group were documented. After all the mice were sacrificed, the tumors in each group were excised and weighed. The body mass changes of mice in each group were calculated as weight at sacrifice/natural death with tumor)−(weight of mice before modeling).

### 2.3. Calculating Tumor Volumes and Inhibition Rates

Tumor volumes were measured every 2 days for 3 weeks by recording the longest diameter (*d*) and maximum vertical diameter (*vd*) of the tumors. The following formula was used to calculate the tumor size: volume = 1/2 × *d* × *vd*^2^. Three weeks after inoculation, the mice were sacrificed by cervical dislocation, and the tumors were removed, cleaned with distilled water, dried with absorbent paper, and weighed. The average tumor weights in the model (M) and experimental (E) groups were calculated; tumor inhibition rates (IRs) in each group were calculated as IR (%) = (M−E)/M × 100%.

BALB/c mice were grouped, inoculated with H22 suspension, and administered as aforementioned. The death dates of mice in each group were recorded. Mice in the blank group were sacrificed the day after any tumor-bearing mice died naturally.

### 2.4. Hematoxylin and Eosin Staining

Tumor tissues were fixed in 10% formalin for 24 h and embedded in paraffin. The tissues were then cut into 3 *μ*m thick slices, incubated at 65°C for 1 h, and subjected to hematoxylin (0.5%, 5 min) and eosin (1%, 1 min) staining at room temperature.

### 2.5. Standardization of Plant Extract

Liquid chromatography mass spectrometry was used to analyze the main components of aconite. To control the quality of aconite, the fingerprint spectrum was established by the ultra-performance liquid chromatography high-resolution mass spectrometry (UHPLC-HRMS) method. These analyses were performed with a Thermo Q Exactive (Thermo Fisher Scientific, Waltham, MA, USA) equipped with a quaternary gradient pump, an autosampler, and an orbitrap mass spectrometer detector. The components were eluted with a gradient system consisting of acetonitrile (I) and aqueous 0.1% formic acid (II) (time, min/II%: 0/95, 2/95, 5/80, 27/10, 35/5, 35.1/95, 40/95). The mass spectrometer scan range was set in the range of 120–1200 m/z. The chromatographic column was a Thermo Syncronis C18 (150 × 2.1 mm, 3 *μ*m; Thermo Fisher Scientific). The mobile phase flow rate was 1 mL/min, and the column temperature was maintained at 45°C.

### 2.6. Flow Cytometry

Flow cytometry was used to detect the number of NK cells and the expression of the NK cell receptors (NKp46, NKG2D, and TIGIT) in peripheral blood and spleen samples, the expression of CD107a in the spleen, and the number of B and T lymphocytes in peripheral blood from each group. Briefly, freshly isolated splenocytes and peripheral blood samples were prepared. Red blood cell lysate was added to cell suspensions for lysis, and samples were centrifuged at 400 × *g* for 5 min, and then washed twice with PBS. The samples were incubated with CD49b-APC antibodies in the dark at 4°C for 30 min and then washed with PBS buffer once. Cells were resuspended in 250 *μ*L PBS for detection on the flow cytometer (FACSCalibur, BD Biosciences, Franklin Lakes, NJ, USA).

### 2.7. Enzyme-Linked Immunosorbent Assay (ELISA)

ELISA was used to detect the expression levels of tumor necrosis factor (TNF)-*α*, interleukin (IL)-1*β*, and interferon (IFN)-*γ* in serum. Briefly, the wells of ELISA plates were coated with samples at an appropriate concentration and then incubated in the dark at 37°C for 90 min. Next, the solution was diluted with double-distilled water, gently shaken for 30 s, and then repeated cleaned three times. Then, 100 *μ*L of biotinylated secondary antibody working solution and incubated with the samples in the dark at 37°C for 1 h. Then, 100 *μ*L of streptomycin HRP working solution was added and incubated in the dark at 37°C for 30 min. Next, 100 *μ*L of chromogen was added and incubated at 37°C for 30 min. Finally, the termination solution was added when there was an obvious color gradient. The absorbance at 450 nm was detected with a Bio-Rad model 680 microplate reader (Bio-Rad, Hercules, CA, USA).

### 2.8. Statistical Analysis

Statistical analyses were performed using SPSS software (version 20.0; IBM Corp., Armonk, NY, USA). The data are expressed as mean ± standard deviation. Significant differences between groups were analyzed by one-way analysis of variance, or the Student's *t*-test. A two-way analysis of variance was applied to analyze significant differences between two groups affected by time. *P* < 0.05 was considered to represent a significant difference.

## 3. Results

### 3.1. Qualitative Test and Phytochemical Screening of Aconite Extract

UHPLC-HRMS results showed the chemical composition of aconite extract. Several compounds, including isotalatizidine, songorine, and hokbusine B, were identified from aconite extract. The compounds and corresponding retention times are shown in Table 1.

### 3.2. The Effect of Aconite on Tumor Growth and Survival Time

During aconite administration, no toxic reactions or abnormal behaviors were found in any group. The subcutaneous tumors decreased body mass in each group, which can be attributed to tumor progression, and aconite alleviated this weight loss. Compared with the *M* group, mice in the MD and HD groups (*P* < 0.05 for MD and *P* < 0.01 for HD) gained significant weight (Table 2).

**Figure 2:**
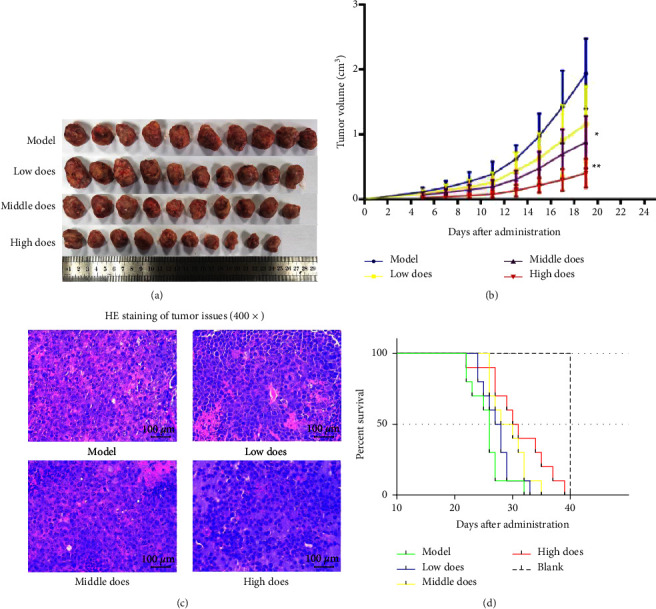
Tumor volumes, tumor histology, and the survival of tumor-bearing mice treated with aconite. (a) Images of tumors excised after 3 weeks of treatment (right) and tumor growth curves (left). (b) Changes in tumor volume. (c) Hematoxylin and eosin staining of tumor tissues. (d) The survival of tumor-bearing mice. Data are presented as mean ± standard deviation; ^*∗*^*P* < 0.05, ^*∗∗*^*P* < 0.01, compared with the model group.

After 3 weeks of treatment, the mice were euthanized, and tumor tissues were resected and weighed. As shown in Figure 2(a), tumor sizes were decreased in the aconite groups in a dose-dependent manner (*P* > 0.05 for LD *vs.* blank; *P* < 0.05 for MD *vs.* blank; and *P* < 0.01 for HD *vs.* blank). The IR in each group was 13.44% (LD), 28.63% (MD), and 42.17% (HD).  Figure 2(b) shows changes in the subcutaneous tumor volumes. Compared with the M group, mice treated with different doses of aconite presented with smaller tumor volumes. Mice in the M group developed grossly visible tumors at the site of injection 4 d after inoculation. Visible tumors developed in 6 d in the LD and MD groups and in 7 d in the HD group. The slower growth of tumors in the MD and HD groups was significant (*P* < 0.05 for MD *vs.* M and *P* < 0.01 for HD *vs.* M). Hematoxylin and eosin staining of tumor issues (400×) showed that, compared with the M group, poorer blood supply and greater tumor necrosis were present in the aconite groups, with the morphology of tumor tissues visibly destroyed (Figure 2(c)). As shown in Figure 2(d), aconite prolonged the survival of tumor-bearing mice. The medium survival time in each group was as follows: M: 26 d (SD: 0.48), LD: 27 d (SD: 1.05), MD: 28 d (SD: 2.37), and HD: 30 d (SD: 1.58). Thus, aconite also prolonged survival time in a dose-dependent manner (*P*=0.005).

### 3.3. Aconite Strengthened Adaptive Tumor Immunity

**Figure 3:**
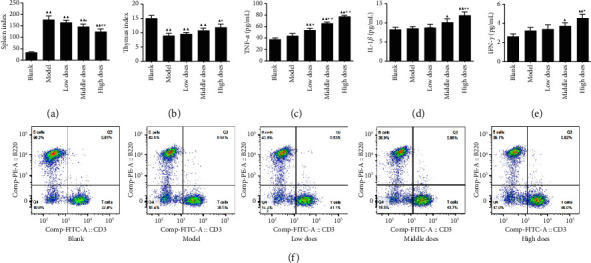
The effect of aconite on adaptive tumor immunity in tumor-bearing mice. (a) Spleen index. (b) Thymus index. (c) Serum TNF-*α*. (d) Serum IL-1*β*. (e) Serum IFN-*γ*. (f) Peripheral T and B cells. ^*∗*^*P* < 0.05, ^*∗∗*^*P* < 0.01 compared with the model group; ^▲^*P* < 0.05, ^▲▲^*P* < 0.01 compared with the blank group.

First, the spleen and thymus indices of each group were calculated. The spleen index of each H22-bearing group was significantly higher than the blank group, while the thymus index was lower. Compared with *M*, the spleen index of aconite-treated mice presented a dose-dependent decrease, while the thymus index steadily increased (Figure 3(a) and 3(b)). Aconite also affected levels of serum cytokines that lead to an immune-favored environment. As shown in Figure 3(c)–3(e), compared with the M group, aconite increased serum levels of TNF-*α*, IL-1*β*, and IFN-*γ* in a significantly dose-dependent manner. The effect of aconite on total B cells and T cells in peripheral blood was also tested. As shown in Figure 3(f), peripheral B cell numbers in tumor-bearing mice were significantly decreased compared with the blank group, indicating that aconite did not have significant effects on the total number of B cells. The numbers of peripheral T cells in tumor-bearing mice were higher than those in the nontumor group, indicating that high-dose aconite treatment significantly upregulated peripheral T cells.

### 3.4. Aconite Enhanced the Quantity and Activity of NK Cells

**Figure 4:**
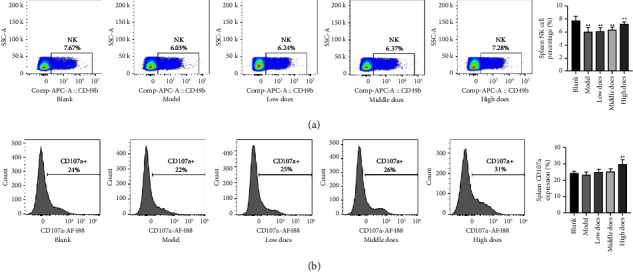
The effect of aconite on splenic natural killer (NK) cell proportions and activity. (a) Proportion of NK cells. (b) CD107a as a marker of NK cytotoxicity. ^*∗*^*P* < 0.05, ^*∗∗*^*P* < 0.01 compared with the model group; ^▲^*P* < 0.05, ^▲▲^*P* < 0.01 compared with the blank group.

As shown in Figure 4(a), the proportion of NK cells in the spleen of tumor-bearing mice was lower than that in the blank group, while high-dose aconite treatment significantly increased the NK cell percentage. The splenic receptor CD107a is a potent biomarker of NK cell cytotoxicity. In this study, only high-dose aconite increased the expression of CD107a in the spleen (Figure 4(b)).

**Figure 5:**
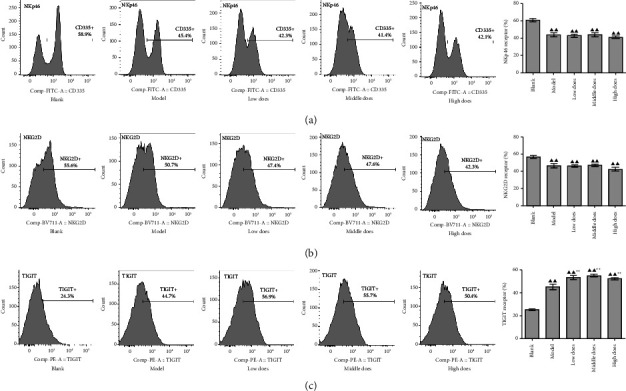
The effect of aconite on natural killer receptor expression in the spleen. (a) NKp46. (b) NKG2D. (c) TIGIT. ^*∗∗*^*P* < 0.01, compared with the model group; ^▲▲^*P* < 0.01 compared with the blank group.

We also investigated whether aconite affected the expression of several receptors on NK cells to perform its immune-enhancing effect. In the spleen, NKp46 (CD335) and NKG2D levels were significantly reduced in tumor-bearing mice, while aconite had no effect on either (Figure 5). TIGIT expression was upregulated in tumor-bearing mice, and aconite (all doses) significantly increased its expression.

**Figure 6:**
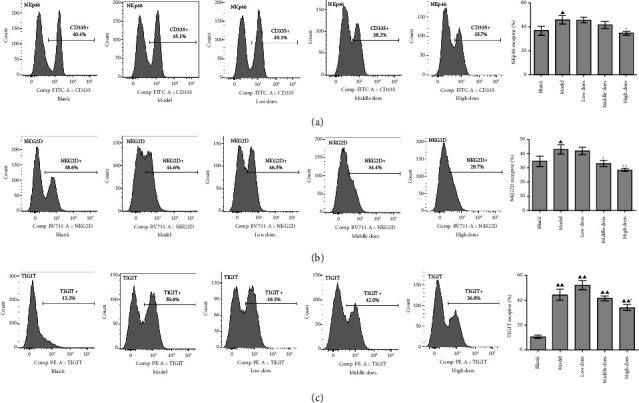
The effect of aconite on peripheral natural killer cell receptor expression. (a) NKp46. (b) NKG2D. (c) TIGIT. ^*∗*^*P* < 0.05, ^*∗∗*^*P* < 0.01, compared with the model group. ^▲^*P* < 0.05, ^▲▲^*P* < 0.01, compared with the blank group.

In peripheral blood, NKp46, NKG2D, and TIGIT expression were significantly upregulated in tumor-bearing mice, while high doses of aconite treatment downregulated the expression of all three receptors (Figure 6).

## 4. Discussion

Several studies have shown that aconite and its main components can suppress the progression of HCC through multiple pathways [7–9, 11]. Taipeinine A, which is derived from aconite, has been found to induce apoptosis in the HCC cell line HepG2 [9]. Aconitum polysaccharide can exert antitumor effects by inducing apoptosis of H22 cells, which is mediated by pituitary tumor transforming gene 1 (PTTG1)-related suppression of P13K/Akt signaling and activation of the downstream MAPK signaling pathway [12]. Aconitine has been proven to inhibit the proliferation of HCC by activating the production of reactive oxygen species, which leads to an increased release of cytochrome *c* from mitochondria and the activation of apoptosis [7]. Nevertheless, few studies on the immunoregulatory effect of aconite in HCC have been reported. In this study, aconite was found to inhibit H22 tumor growth in vivo and to alleviate the body weight reduction effects of subcutaneous tumors. Meanwhile, indices of adaptive tumor immunity status were improved in tumor-bearing mice, with the thymus index and IFN-*γ* significantly upregulated. Strangely, TNF-*α* and IL-1*β* are extensively regarded as immunosuppressive cytokines and both were unexpectedly upregulated following aconite treatment, which may be attributed to M2-polarized macrophages. This unexpected phenomenon requires further experimental validation.

NK cells are a significant component of the nonspecific immune response that exert antitumor effects by both direct cytolytic activity and the secretion of immunostimulatory cytokines [13–16]. Studies have confirmed that the occurrence of many tumors is related to the loss of the recognition function of NK cells, which is also one of the main mechanisms of tumor immune evasion [17–20]. The release of lytic granules containing granzymes and perforin mediates the cytolytic activity of NK cells via binding death receptors on tumor cells, and this can be demonstrated by the expression of CD107a on the surface of NK cells. The expression of CD107a can also be evaluated as a marker of the activation of effector NK cells [21–23]. Innovatively, we found that aconite treatment enhanced NK-mediated immune responses by increasing both the quantity and activity of NK cells. In this study, aconite was proven to be effective in increasing the NK cell percentage in the spleen. Moreover, the expression of CD107a in the spleen could be upregulated under high-dose aconite treatment, which indicated the direct oncolytic activity of NK cells. Activated NK cells can also release TNF-*α* [24]; however, the role of TNF-*α* signaling in HCC progression remains controversial. For one thing, TNF-*α* can inhibit tumors via activating programmed death cascades in targeted cells, with devastating effects on the function of lysosomal enzymes and/or lysosomal membrane integrity. TNF-*α* signaling can also be used to induce B7-H1 and PD-L1 expression on CD8^+^ T cells via several pathways, thereby causing immune resistance [25–27]. We found that three different doses of aconite all increased TNF-*α* levels in the serum of tumor-bearing mice. The activation and proliferation of NK cells mediated by aconite treatment may be responsible for the release of TNF-*α*.

The functions of NK cells depend on the regulation of activated receptors and inhibitory receptors expressed on their surfaces. The former are represented by the NCR family (NKp30, NKp44, NKp46, and NKp80), NKG2D, NKG2C, CD226 (DNAM-1), 2B4, and CD16, while the latter mainly includes TIGIT, KIRs, CD96, and CD94/NKG2A [28, 29]. NKp46 is involved in the killing and activation of NK cells against pathogens, tumor cells, virus-infected cells, and their own cells [30]. NKG2D is currently recognized as an activated receptor that plays a key role in antitumor immune responses and is also a key target for tumor immune escape [31]. TIGIT can restrict the proliferation of T cells, inhibit the activation of T cells and NK cells, and block multiple steps of the cancer immune cycle [32]. In this study, we found that inhibitory receptors on NK cells such as TIGIT were significantly downregulated in peripheral blood following high-dose aconite treatment, thus promoting the activation of NK cells. The increased secretion of IFN-*γ* may also be related to this change. To our surprise, TIGIT was upregulated in the spleen following aconite treatment. This may be related to the increased expression of CD155, the shared ligand of TIGIT and CD226 [33, 34]. The activated receptor CD226 plays a dominant role, thus increasing the quantity and activity of NK cells. Nevertheless, this hypothesis requires further experimental validation.

**Figure 7:**
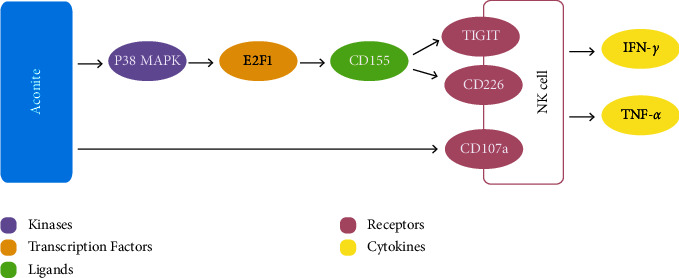
The possible mechanism through which aconite treatment modulates adaptive immunity and NK cell-mediated immunity.

Aconite and its components have been found to conduct bidirectional regulation of immune status in diseases, including chronic glomerulonephritis, rheumatic arthritis, osteoarthritis, and allergic rhinitis [35–37]. This effect can be explained by both immune stimulation and anti-inflammation effects. On the one hand, aconite upregulates CD4^+^/CD8^+^ T cells and stimulates related cytokines, including IL-4, IL-10, and IFN-*γ* in patients with septicopyemia [35]. As for cancerous diseases, aconite can also stimulate tumor immunity in patients with breast cancer undergoing chemotherapy, which is mediated by the upregulation of CD4^+^/CD8^+^ T cells and NK cells [38]. On the other hand, aconite can inhibit the release of inflammatory cytokines, including IL-6, IL-8, and IL-10, in several autoimmune diseases [39, 40]. Our study found that aconite had an immunomodulatory effect on H22 tumor-bearing mice. Combined with the previous research of our group and relevant literature, we speculate that aconite may enhance adaptive immunity and NK cell-mediated immunity by regulating the secretion of immunostimulatory cytokines, upregulating T cells and NK cells, and altering the expression of CD107a and NK receptors. The deep mechanism may be related to the p38 MAPK signal transduction pathway, which could activate the E2F1 transcription factor and upregulate the expression of ligand CD155 (Figure 7). The specific mechanism and main functional component still need further study.

## 5. Conclusions

The results of our study showed that aconite had a therapeutic effect on HCC subcutaneous tumor-bearing mice, which could be attributed to the immunoregulatory effect of aconite, including adaptive immunity and NK-mediated immunity. This study revealed a novel mechanism through which aconite exerts an antitumor effect, not only through apoptosis induction pathways. Considering the high incidence and poor prognosis of HCC patients, our findings could be of valuable clinical significance in the treatment of HCC. However, the bidirectional regulation of adaptive immunity mediated by aconite as well as the deep mechanism through which aconite activates NK cells still require further investigation. These studies may provide more sound evidence before the introduction of this treatment into routine clinical practice.

## Figures and Tables

**Figure 1:**
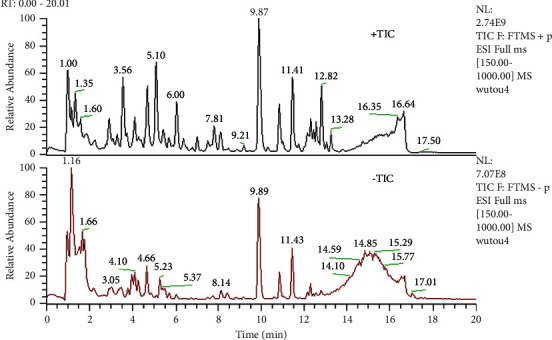
The total ion chromatogram (TIC) profile of aconite extract.

**Table 1:** Chemical compounds in aconite extract as analyzed by ultra-performance liquid chromatography high-resolution mass spectrometry.

Note	Retention time (min)	Chemical compounds	Molecular formula	Molecular weight
1	3.56	Isotalatizidine	C23H37NO5	407.54362
2	4.12	Songorine	C22H31NO3	357.48648
3	4.68	Hokbusine B	C27H35NO5	453.57054
4	5.10	Neoline	C24H39NO6	437.5696
5	6.00	Talatisamine	C24H39NO5	421.5702
6	7.81	14-Acetyltalatisamine	C26H41NO6	463.60688
7	9.87	Benzoylmesaconine	C31H43NO10	589.67386
8	10.83	Benzoylaconitine	C32H45NO10	603.70044
9	11.41	Benzoylhypacoitine	C31H43NO9	573.67446
10	12.25	Aconitine	C34H47NO11	645.73712
11	13.04	Hypaconitine	C34H47NO10	629.73772

The concentrations of safflomin A, benzoylmesaconine, benzoylaconitine, benzoylhypacoitine, aconitine, and hypaconitine were detected by the UHPLC-HRMS method and were 4.1 mg/g, 5.63 mg/g, 6.45 mg/g, 1.22 mg/g, and 1.35 mg/g, respectively, in the extracts. The chromatographic profile of the extract is shown in Figure 1.

**Table 2:** Effect of aconite on body weight in mice with subcutaneous liver tumors.

Group	Weight before administration (g)	Weight after tumor resection (g)	Body weight change (g)
Blank	18.19 ± 0.58	25.47 ± 0.72	7.28 ± 0.36
Model	18.35 ± 0.83	22.34 ± 1.33^▲▲^	3.99 ± 0.94^▲▲^
LD	17.99 ± 0.58	22.85 ± 0.74^▲▲^	4.86 ± 0.89^▲▲^
MD	18.00 ± 0.82	23.39 ± 1.28^▲▲^	5.39 ± 1.07^▲▲^^*∗*^
HD	17.61 ± 0.53	23.99 ± 1.86^▲^^*∗∗*^	6.38 ± 2.02^*∗∗*^

Data are presented as mean ± standard deviation. ^▲^*P* < 0.05, ^▲▲^*P* < 0.01, compared with the blank group; ^*∗*^*P* < 0.05, ^*∗∗*^*P* < 0.01, compared with the model group.

## Data Availability

The datasets used and/or analyzed during the current study are available from the corresponding author on reasonable request.
